# Public image of nursing in modern society: An evolving concept analysis

**DOI:** 10.1002/nop2.70033

**Published:** 2024-09-16

**Authors:** Ying Duan, Xianqiong Feng, Hengyi Xiao

**Affiliations:** ^1^ Department of Nursing West China Hospital, Sichuan University/West China School of Nursing, Sichuan University Chengdu China; ^2^ Department of Nursing Affiliated Hospital of North Sichuan Medical College Nanchong China; ^3^ Lab for Aging Research National Clinical Research Center for Geriatrics State Key Laboratory of Biotherapy Chengdu China; ^4^ Department of Geriatrics West China Hospital, Sichuan University Chengdu China

**Keywords:** concept analysis, image, nursing, public

## Abstract

**Aims:**

This study aimed to analyse the evolution of the public image of nursing in the context of the constantly developing nursing profession.

**Design:**

The Rodger's evolving concept analysis was applied.

**Methods:**

PubMed, CINAHL, Web of Science, Scopus, and ProQuest databases were searched for articles published between 1 January 2001, and 30 April 2022, using the search terms; “NURS * AND image”. The selected literature was screened using Rodgers' evolutionary method to explore the attributes, antecedents and consequences of the concept.

**Results:**

The defining attributes were identified as nursing (nursing as the collective object), public (public as the collective subject) and information (the medium of interaction between the collective subject and the collective object). Nursing elements were classified into intrinsic elements (professional spirit, professional knowledge and professional skills) and extrinsic (appearance, language and behaviour) elements. Public elements were further subcategorized into public categories (internal organizational public and external organizational public) and public perceptions (cognition, emotion and behavioural intention). The information elements are mainly classified as information generation, dissemination, identification, processing and judgement. The antecedents and consequences of the public perception of nursing were also identified.

**Conclusions:**

The public image of nursing is dynamic and has evolved over time. Its dynamism and malleability imply that the traditional public image of nursing can be improved through targeted interventions in nursing practice, management and education.

**Implications for the Profession:**

Identifying the antecedents and consequences associated with the public image of nursing will help the healthcare organizations adopt effective strategies to alleviate the shortage of the nursing workforce and promote the development of the nursing profession.

No Patient or Public Contribution.

## 1 INTRODUCTION

The public image of nursing has been a topic of concern because it is not only related to the shortage of the nursing workforce (Ndirangu et al., 2021; Somers et al., 2010; Squires et al., 2019), but also affects the development of nursing profession (López‐Verdugo et al., 2021; van der Cingel & Brouwer, 2021). Traditional stereotypes are still dominant in the media and the public, for example, nursing is often portrayed in the media as feminine and caring (Elmorshedy et al., 2020; Stanley, 2008), but these images are inaccurate and can have a negative impact on the recruitment of nurses (Morris‐Thompson et al., 2011). Such biases, they may discourage potential students, particularly men, from pursuing a career in nursing (Morris‐Thompson et al., 2011). Additionally, the public tends to perceive nurses as being subordinate to physicians (Price et al., 2013), as nurses do not require higher levels of education (Hadid & Khatib, 2015; Hoeve et al., 2014) and advanced nursing techniques (Hadid & Khatib, 2015). These perceptions may eventually reduce a decline in nurses' self‐esteem and professional identity (Hoeve et al., 2014) and increase their willingness to leave the profession (Elmorshedy et al., 2020; Takase et al., 2006).

There are ongoing efforts to address the issue of professional image in the nursing industry. Consequently, multiple strategies have been developed to improve public perceptions about the nursing profession (Auker, 2004; Emeghebo, 2006), and some of the typical examples have been reported in the literature are as follows: ‘Truth in Nursing’, is a non‐profit organization dedicated to raising public awareness of the role of nurses (Geller & Summers, 2014). It conducts educational training programmes for nurses to improve their professional competence (Toren et al., 2011), and organises hospital visits for senior high school students as educational summer camps to elicit their interest in nursing (Drenkard et al., 2002; Gomez & Brostoff, 2018). The Johns Hopkins School of Nursing in the United States produces and disseminates nursing advocacy videos through social media to communicate the goals and scope of nursing work (Kress et al., 2018). As a result, there has been an increase in people's positive attitudes towards nursing (Ali et al., 2021; Donelan et al., 2008; Kalisch et al., 2007). A survey by Kalisch et al. (2007) on the media image of nurses showed that approximately 70% of internet sites considered nurses to be intelligent and educated, and 60% considered nurses to be respected, competent, and trustworthy. A questionnaire survey in Pakistan also revealed that 70% of the participants believed that nursing is a respectable profession and 88.5% of the participants believed that the nursing profession has a good future (Ali et al., 2021). Although an increasing number of people appreciate nursing, it is still not viewed as an attractive career (Ali et al., 2021; Meiring & van Wyk, 2013; Ndirangu et al., 2021), and the shortage of the nursing workforce has been largely remained unchanged (Ndirangu et al., 2021), possibly due to the public's perceptions and opinions regarding nursing (Ndirangu et al., 2021). Professions that serve the public must carefully consider how people perceive and evaluate them because public perception not only affects the service delivery of professionals (López‐Verdugo et al., 2021; Valiee et al., 2020), but also influences their recognition by the state, professional organizations and the public. Therefore, establishing a positive public image for nursing is an urgent issue that must be addressed to create an empowered nursing workforce.

Establishing a positive public image of nursing primarily requires a clear understanding of the concept of public image. Current definitions of this concept are diverse and are based on different perspectives. Milisen et al. (2010) state that the public image of nursing constitutes a collection of ideas, principles, perceptions, expectations, and experiences of people outside the nursing profession, especially the recipients of nursing services. Squires et al. (2019) define the public image of nursing as the perception of the entire nursing profession by ‘the public’, which includes people interacting with nurses. An extensive review of the literature revealed that the current understanding of “public” is vague and confusing. Valiee et al.'s (2020) study interpret the public as a non‐nursing group. Although this classification reveals the exclusivity of the public image, it is general and ignores the different views and assessments of individuals who interact closely with nurses. Adopting this definition may lead to obstacles in survey research, targeted organization assessment, and the formulation of targeted intervention strategies for different categories of the public. In some studies, nursing students are not considered a part of the nursing community (Ward, 2006), however, in practice, their behaviour indirectly affects people's views about the nursing profession (De gagne et al., 2019). Therefore, a clear definition of the public and different nursing groups is warranted through concept analysis.

However, the public's perceptions of nursing frequently fail to match the reality of the profession. In addition, empathy and willingness to make sacrifices are emphasized as the main characteristics of nurses when proclaiming a nursing profession. Thus, interventions designed to increase knowledge of nursing characteristics can enhance the public's understanding of the nursing profession Ward (2006).adopted Rodgers' evolutionary method to analyse the attributes of the image of nursing and obtained six components: care, attitude, knowledge, behaviour, autonomy, and uniformity. However, the nursing profession has evolved rapidly since the beginning of the twenty‐first century. The workplace, nature of work, role, and scope of nursing are now considerably enriched and broad, resulting in a diverse and complex nursing profession. As such, there is a need to update the characteristics of nursing. An Iranian scholar Rezaei‐Adaryani et al. (2012) developed a schematic model for understanding the nursing image by conducting an evolutionary concept analysis of antecedents, attributes and consequences. The attributes of the nursing image encompass four domains: the public's perception of nursing image, self‐image of nursing, nurses' perception of the public image, and nursing image portrayed in the media. Furthermore, the concept of nursing image is multidimensional, all‐inclusive, paradoxical, dynamic and complex. However, existing study only indicated the categories of nursing image and did not analyse the components of the nursing public image. Consequently, there is a lack of understanding of the characteristics and attributes of the concept of nursing image from the perceptive of public perception.

Studies have demonstrated the high social awareness and reputation of nursing during public health emergencies and natural disaster events (Donelan et al., 2008; Hall et al., 2003; van der Cingel & Brouwer, 2021). Currently, nurses worldwide are contributing significantly in the response to COVID‐19 pandemic. It is necessary to clarify the connotations of the nursing public image through concept analysis and identify the influencing factors for a better understanding of the nursing image.

### 1.1 Aims

This study aimed to analyse the evolution of the public image of nursing in the context of a constantly developing nursing profession.

## 2 METHODS

### 2.1 Rodger's method

Given that the public image of nursing changes over time with the development of 调the nursing profession and the changes in the healthcare system, Rogers' evolutionary method was adopted in this study to clarify the concept of public image of nursing and its current usage. The process of delineating the attributes, antecedents, and consequences of concepts is a fundamental aspect of Rogers' analytical methodology (Rodgers & Knafl, 2000). Defining attributes, which refer to the characteristics, elements, or components of a concept, enhances comprehension of the concept and differentiates it from other similar concepts. Antecedents are traits that precede or lead to a conceptual events or experiences. The outcomes are the results of applying the concept in a real‐life situation (Li et al., 2018).

The study included the following systematic steps: (1) the public image of nursing was determined as a concept to be analysed; (2) key databases were selected and a retrieval strategy was defined through pre‐retrieval; (3) conceptual attributes were analysed; (4) antecedents and consequences were analysed; (5) model cases that fit the concept were provided (if needed), and (6) hypotheses were verified and their applications to promote the evolution of the concept were examined (Rodgers & Knafl, 2000).

### 2.2 Data source

Five databases PubMed, CINAHL, Web of Science, Scopus, and ProQuest were searched. Initially, we comprehensively explored the scope of the study regarding the nursing image using the terms “nurs*” and “image” in the titles/abstracts/subject of the studies published from 1 January 2001, to 30 April 2022. The search strategy for each database is presented in Appendix 1.

Journals articles, books and dissertations published in English that describe the attributes or, antecedents, and consequences of the public image of nursing were included.

The exclusion criteria were conference papers, comments, letters, interviews, news, viewpoints, book reviews, articles not available as full‐text publications and articles only mentioning the study terms without an explanation.

**FIGURE 1:**
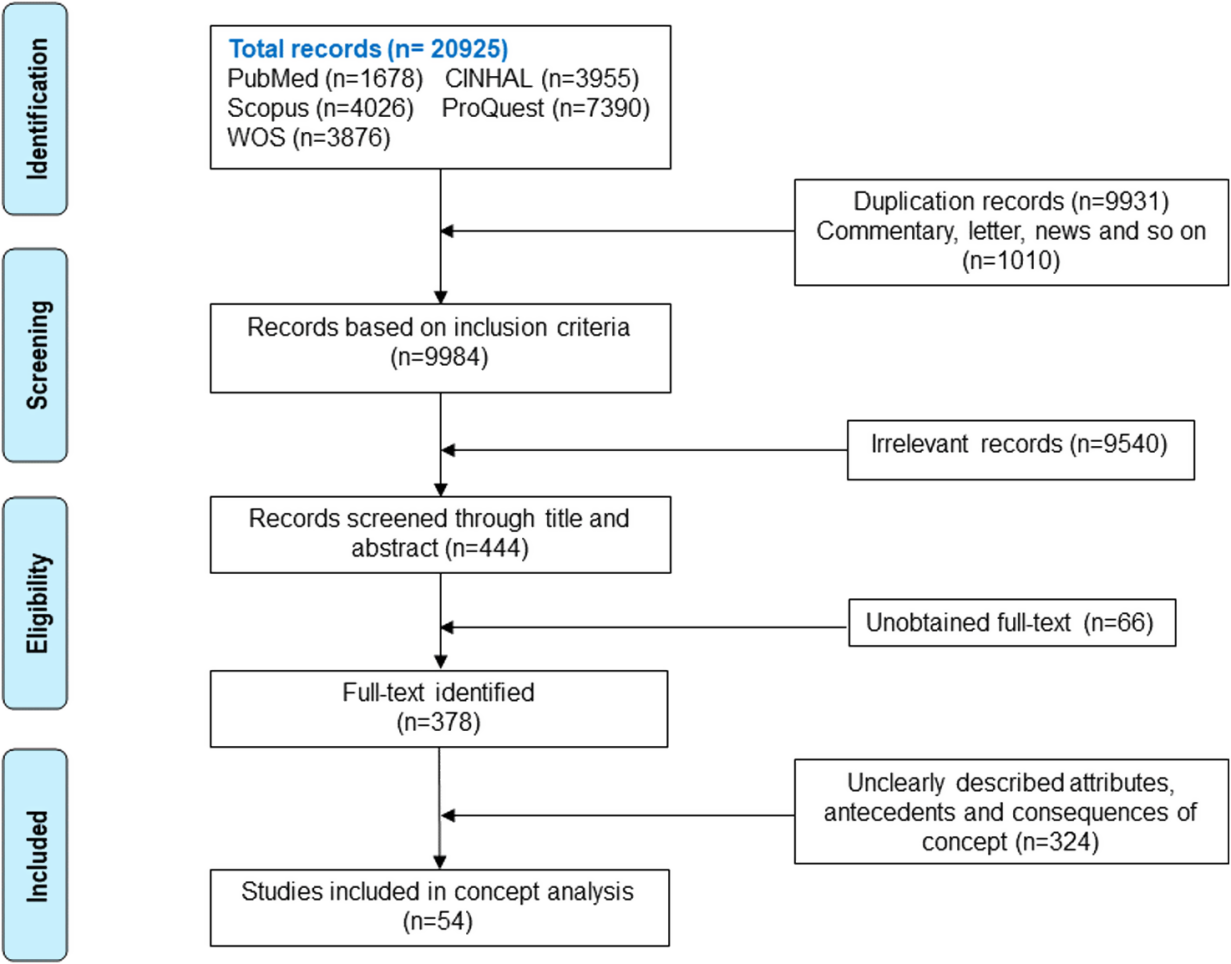
Flow chart of literature search and screening.

The literature review process included the following five phases: (1) Original publications were searched in the electronic databases using the established search strategies and imported into Endnote7.0 for further appraisal and management. (2)The title, abstract, keywords, and subject of each publication were reviewed, and preliminary screening was conducted according to the inclusion and exclusion criteria. (3) Full texts of the publications were reviewed for evaluation. (4) Full texts were analysed more deeply to identify core information and for detailed evaluation. (5) Reference lists of all included studies were scanned to identify additionally potentially relevant studies. A flowchart of the literature screening process is shown in Figure 1.

### 2.3 Data analysis

After the initial review of the included articles, we coded the key information. Themes were then identified using Roger's method. The extracted data were placed in an Excel sheet and a thematic approach was conducted to categorize antecedents, attributes, and consequences of the public image of nursing. The two reviewers conducted in‐depth analyses and discussions to determine the conceptual categories and their hierarchical structures.

## 3 RESULTS

A total of 54 publications were included in this study, including 50 journal articles, 3 dissertations and 1 book.

### 3.1 Identifying the public image of nursing

The first study of the public image of nursing was conducted by the American Nurses Association in 1940 (Simmons, 1962). Research in this area has focused primarily on three aspects. The first was to directly survey the image of nursing as perceived by the public, including citizens, physicians, and other health professionals such as children, middle school students, high school students, medical college students, career counsellors and tutors (Hadid & Khatib, 2015; King et al., 2007; Marcinowicz et al., 2009; Meiring & van Wyk, 2013; Mert et al., 2020; Morris‐Thompson et al., 2011; Neilson & McNally, 2013; Williams et al., 2018; Williams & Dickstein‐Fischer, 2019). The second aspect was to indirectly understand the public's view of nursing based on media portrayal of the image of nursing (Chapman, 1977; Emeghebo, 2006). Kalisch, an American scholar, performed a series of extensive studies on nursing image representation in the media since the 1980s. These studies analysed the gender role/performance of nurses in mass media (TV, books and movies) and identified four stereotypes of nurses in different time periods: angel, handmaiden, battleaxe and sexy (Kalisch & Kalisch, 1982a, 1982b, 1982c). Subsequently, many researchers analysed nursing images in media advertisements, networks and social media (Cohen & Bartholomew, 2008; Kalisch et al., 2007; Kelly et al., 2012; Koo & Lin, 2016; Şahan et al., 2021). The third aspect was nurses' and nursing students' perceptions of nursing, which was explored by examining the relationship between the variables related to nurses' work (Karanikola et al., 2011; Rahman & Shousha, 2013; Rubbi et al., 2019; Weaver et al., 2013), such as professional identity, job performance, job satisfaction, and turnover intention (Emeghebo, 2006; Takase et al., 2002; Takase et al., 2006).

Overall, the concept of nursing public image has not been adequately elaborated in the literature. However, consistently across publications, this concept has been defined as a collection of perceptions, principles, expectations and experiences of care by people outside the nursing profession, particularly the recipients of nursing services (Milisen et al., 2010). This concept highlights that public perception of nursing is a psychological process that requires a comprehensive judgement of nursing impressions according to personal experience, values, and expectations.

### 3.2 Concept analysis

**FIGURE 2:**
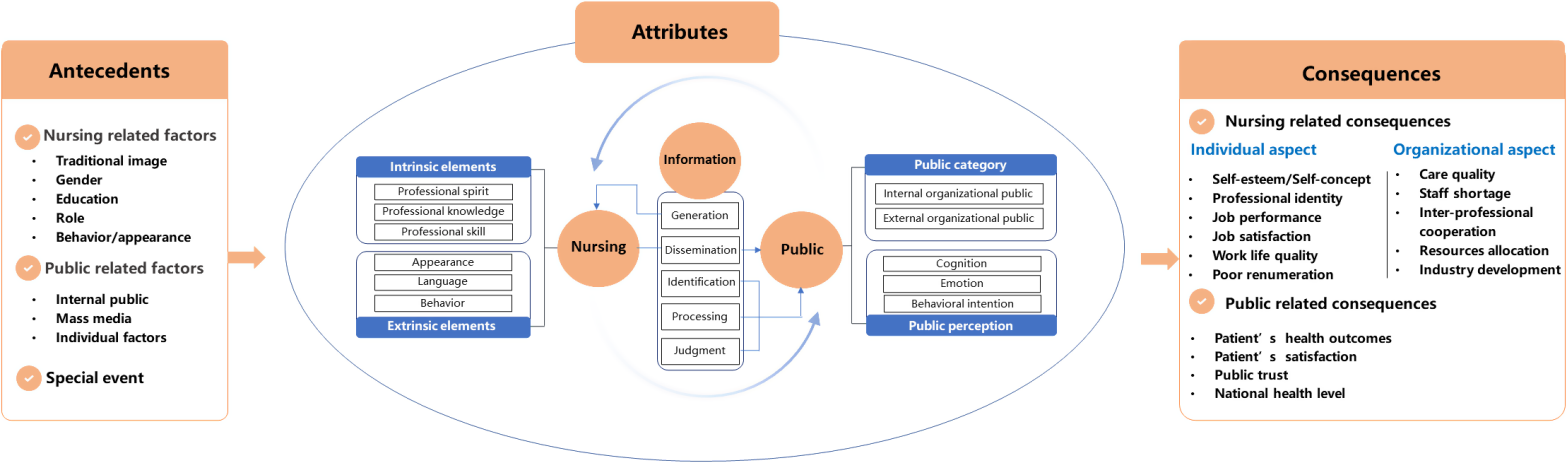
The conceptual framework of public image of nursing.

The antecedents, attributes and consequences of the public image of nursing were identified using thematic analysis of the included publications. The details of each study are listed in Table 1. Figure 2 depicts the conceptual framework of the public image of nursing.

**TABLE 1:** Results of a thematic analysis with included citations (*n* = 54).

Author/Year	Country	Study design	Antecedents nursing antecedent (NA) public antecedents (PA) special event antecedents (SEA)	Attributes object attributes (OA) subject attributes (SA) information attributes (IA)	Consequences nursing consequences (NC) public consequences (PC)
Takase et al. (2002)	Australia	Quantitative study			NC
Auker (2004)	USA	Discourse analysis	NA, PA	SA	NC
Emeghebo (2006)	USA	Qualitative study	NA, PA	OA, SA	
Takase et al. (2006)	Australia	Quantitative study			NC
Tzeng (2006)	Taiwan, China	Quantitative study	NA, SEA		NC
Ward (2006)	USA	Quantitative study	NA	OA	
Fletcher (2007)	Canada	Literature review		OA, SA	
Kalisch et al. (2007)	USA	Content analysis		SA	NC
Donelan et al. (2008)	USA	Quantitative study	PA, SEA	SA, IA	
Cabaniss (2011)	USA	Literature review	PA	OA, IA	NC, PC
Karanikola et al. (2011)	Greece	Qualitative study	NA, PA	OA	NC
Morris‐Thompson et al., (2011	England	Qualitative study	NA, PA	OA, SA, IA	NC
Thomas et al. (2010)	USA	Quantitative study	NA	OA, SA	
Kelly et al. (2012)	England	Critical discourse analysis	NA, PA		NC
Rezaei‐Adaryani et al. (2012)	Iran	Concept analysis	NA, SEA		NC, PC
Varaei et al. (2012)	Iran	Quantitative study	NA, PA	OA, SA	NC, PC
Meiring & van Wyk (2013)	South Africa	Quantitative study	NA, PA	OA, SA, IA	NC
Hoeve et al. (2014)	Netherlands	Literature review	NA, PA	SA	NC
Price & McGillis Hall (2014)	Canada	Literature review	PA	OA	NC
Valizadeh et al. (2014)	Iran	Qualitative study	NA, PA	OA	NC
Weaver et al. (2014)	Australia	Qualitative study	NA	IA	NC
Wocial et al. (2018)	USA	Qualitative study	NA	OA, IA	
Hadid & Khatib (2015)	Israel	Quantitative study	NA, PA	OA, SA	NC
Girvin et al. (2016)	England	Literature review	NA, PA	OA, SA, IA	NC
McGillis Hall & Kashin, (2016)	Canada	Literature review	PA, SEA	OA, SA	
Glerean et al., (2017)	Turkey	Literature review	NA, PA	OA, SA	NC
Lúanaigh (2017)	England	Literature review	NA, PA	OA, SA	NC, PC
Rubbi et al. (2017)	Italy	Quantitative study	PA	IA	NC, PC
Daigle (2018)	USA	Qualitative study	NA	OA, SA	
De gagne et al. (2019)	USA	Date mining analysis	NA	OA	
Glerean et al. (2019)	Finland	Qualitative study	NA, PA	OA	
Squires et al. (2019)	Georgia	Qualitative study	NA, PA	OA, SA, IA	
Afshar et al., (2020)	Iran	Qualitative study	PA	OA	NC
Appiah et al. (2020)	Ghana	Literature review	NA	OA	
Elmorshedy et al. (2020)	Arab	Quantitative study	NA	SA	NC
Godsey et al. (2020)	USA	Qualitative study	NA, PA, SEA	OA, SA, IA	NC, PC
Maliheh et al. (2020)	Iran	Quantitative study	NA, PA	OA, SA, IA	NC, PC
Sahakyan et al. (2020)	Armenia	Mixed method study	NA	SA	NC
Valiee et al. (2020)	Iran	Qualitative study	PA	OA, SA	NC, PC
Ali et al. (2021)	Pakistan	Quantitative study	NA, PA	IA	
Foà et al. (2021)	Italy	Quantitative study	PA, SEA	SA, IA	NC
Gill & Baker (2021)	England	Literature review	NA, PA	IA	NC, PC
López‐Verdugo et al. (2021)	Spain	Literature review	NA	OA, SA	NC, PC
Ndirangu et al. (2021)	Pakistan	Quantitative study	NA, PA		NC
Roshangar et al. (2021)	Iran	Quantitative study	NA, PA, SEA	SA	NC, PC
Şahan et al. (2021)	Turkey	Emotion analysis	PA, SEA	OA, SA, IA	PC
van der Cingel & Brouwer (2021)	Netherlands	Literature review	NA, SEA	OA, SA, IA	NC
Zhang et al. (2021)	China	Quantitative study	SEA		NC
Cao et al. (2022)	China	Picture analysis	NA, SA	OA, IA	NC
Apaydin Cirik et al. (2022)	Turkey	Mixed method study	NA, PA, SEA	OA, SA, IA	NC
Grinberg & Sela (2022)	Israel	Quantitative study	NA, PA		NC
Mohsen et al. (2022)	Spain	Quantitative study	NA, PA	SA	NC
Rodríguez‐Pérez et al. (2022)	Spain	Literature review	PA	OA, SA	
Woldasemayat et al. (2022)	Ethiopia	Quantitative study	PA		NC, PC

### 3.3 Defining attributes

Based on the analysis of the original concept of nursing public image and descriptions in the literature, nursing public image was found to have three elements: nursing (nursing as the collective object), public (public as the collective subject) and information (medium of interaction between the collective subject and the collective object). Initially, the public viewed the nursing profession as the object of their understanding, but through direct and indirect interactions with nursing and information medium, they formed a new impressions, views and perceptions of nursing.

#### 3.3.1 Nursing (nursing as the collective object)

In general, nurses refer to a professional group of individual nurses who are legally engaged in nursing work, either presently or in the future. Nursing is considered a collective object of public perception, objective reality, and the source of public perception. It builds and provides the image of nursing. It comprises both intrinsic and extrinsic elements.

The intrinsic element is the core part of nursing, which is composed of the knowledge, skills, and ideological dimensions of the people involved in nursing work. Many studies have identified professional knowledge, skills and care as are essential attributes of the nursing profession (Fletcher, 2007; Karanikola et al., 2011; Hoeve et al., 2014; Price & McGillis Hall, 2014; Valizadeh et al., 2014; López‐Verdugo et al., 2021; Cao et al., 2022; Rodríguez‐Pérez et al., 2022; Afshar et al., 2020; Glerean et al., 2019; Donelan et al., 2008; Meiring & van Wyk, 2013; McGillis Hall & Kashin, 2016; Ward, 2006). We identified and divided intrinsic elements into three dimensions: professional spirit, knowledge and skills.

The nursing professional spirit dimension comprises values, professional ethics, beliefs and attitudes that are considered important in the profession (Godsey et al., 2020; van der Cingel & Brouwer, 2021). It is a professional code for guiding and regulating nurses in nursing practice and its most important connotation reflects the key attributes of nursing. ‘care’, ‘compassion’, ‘morality’, ‘honesty,’ ‘respect’ and ‘autonomy’ are all important nursing characteristics for both nurses and the public (Afshar et al., 2020; Cao et al., 2022; Emeghebo, 2006; Glerean et al., 2017; Morris‐Thompson et al., 2011; Murphy et al., 2009; Thomas et al., 2010; Valiee et al., 2020; Varaei et al., 2012; Ward, 2006; Wocial et al., 2014). In practice, the formation of the nursing image is inseparable from the ideals and values in practice (Fletcher, 2007). The integration of professional spirit into nursing work can guide nurses to face and deal with complex value oriented problems in the clinical environment and influence nurses to fulfil their professional roles and responsibilities to improve the quality of care delivered to patients (López‐Verdugo et al., 2021).

Professional knowledge means that nursing staff must have a complete set of specialised knowledge necessary to deliver quality nursing services, including theoretical, practical and clinical knowledge (Rodríguez‐Pérez et al., 2022; Thomas et al., 2010). Professional knowledge encapsulates the professional attributes and important characteristics of nursing that are essential for the construction of professional identity and nursing image (Emeghebo, 2006; Rodríguez‐Pérez et al., 2022; Ward, 2006). Afshar et al. (2020) believe that specialisation is at the core of the nursing profession. In highly specialised fields, nurses can apply their extensive theoretical and practical knowledge to provide precise clinical judgement and professional decision‐making for the timely satisfaction of patient needs and greatly improve the quality of care provided and professional autonomy (Karanikola et al., 2011; Fealy, 2004).

Professional skills include technical, intellectual, cognitive, organizational and communication (Milisen et al., 2010), and interpersonal skills, and other skill sets involved in nursing practice that are widely used in the process of serving patients (Thomas et al., 2010). Some studies have suggested that professional skills are the most valuable characteristics of nursing effectiveness and a prerequisite for nursing practice (Rodríguez‐Pérez et al., 2022; Valiee et al., 2020). Tzeng (2006) found that professional skills build positive professional images. Hadid and Khatib (2015) state that a positive image of nursing lies in the public's belief that nurses are qualified. Communication skills are especially important in effective nurse–patient interactions, and proper communication and respect positively influence patients' trust in nurses (Rodríguez‐Pérez et al., 2022; Valiee et al., 2020). Interpersonal skills are often the standard for judging the quality of the nursing service (Price & McGillis Hall, 2014).

The extrinsic elements of nursing are the explicit parts perceived by the public through the personal sensory system. Three extrinsic dimensions were identified: appearance, language and behaviour.

Appearance includes the look, dress, posture and movements of nurses. It is a form of nonverbal communication that people use to form judgements and behavioural responses. When interactions occur between two people, they form perceptions of each other's roles in relationships and society based on their appearance (Thomas et al., 2010). Studies have shown that the appearance of nurses is an important predictor of the nursing public image (Albert et al., 2008; Apaydin Cirik et al., 2022; López‐Verdugo et al., 2021; Squires et al., 2019). The professional appearance portrayed by nurses' uniforms embody the role recognition and competency characteristics that convey a professional image of nursing to patients (Wocial et al., 2018; Thomas et al., 2010; Ward, 2006). Historically, white uniforms and dovetail caps worn by nurses represented a classic angelic image (Skorupski & Rea, 2006). Uniform‐style design has remained neutral, with jackets and pants becoming the standard design, and more attention now shifting to comfort and functionality in the late 1990s (Daigle, 2018; Thomas et al., 2010). When nurses remove their hats and wear stethoscopes around their necks, they convey a professional image to the public (Skorupski & Rea, 2006). Studies have shown that the colour and cleanliness of uniforms can affect patients' perceptions of the nursing profession. In addition, Thomas et al. (2010) have reported that nurses' inappropriate professional appearance such as tattoos and piercing of the lips and nose, can lead to patients' perceptions that nurses lack off care, knowledge and skills, reducing public trust in nurses.

Language is also an element that influences the perception of nursing (Emeghebo, 2006). Nursing practitioners in all medical institutions must treat patients with respect and compassion. The words ‘please’ and ‘thank you’ should be routinely used in practice, as respecting and appreciating patients can contribute to improvement in overall health (Appiah et al., 2020).

The image of nursing by society is shaped by the behaviour of nurses (Şahan et al., 2021; Ward, 2006), which includes professional and extra‐professional behaviour (Cabaniss, 2011; Varaei et al., 2012; Emeghebo, 2006). In practice, professional interactions form the basis of professional behaviour (Afshar et al., 2020). Nurses spend most of their shift hours caring for and interacting with patients (Maliheh et al., 2020), especially in emergency environments, which profoundly affects their perceptions of nursing (Godsey et al., 2020). Consequently, messages conveyed through their actions determine the profession's current public image (Girvin et al., 2016; Maliheh et al., 2020). According to a survey conducted by De gagne et al. (2019), cyber‐incivility behaviours by nurses and nursing students on Twitter, such as blasphemy, drunkenness, and racial discrimination, are more visible to the public and may tarnish the image of the profession and violate the code of ethics.

#### 3.3.2 Public (public as the collective subject)

The public functions as a collective subject shaping the perception of nursing and serves as the entity responsible for recognising and appraising the nursing profession. This entity may manifest as an individual, a group, or an organization, intricately linked to, engaging with and influencing on the nursing community.

Based on the level of interaction between the public and the nursing group, the public can be divided into internal organizational public and external organizational public. The internal organizational public refers to nurses' peers (physicians, hospital administrators, health technicians, public health officials and health professions students), patients, and their families (Girvin et al., 2016; Morris‐Thompson et al., 2011; Valiee et al., 2020; Varaei et al., 2012). This kind of public has a more intuitive and accurate perception of the behaviour of nursing staff, as they interact frequently with them in both work and life. The external organizational public includes the general public and media. This kind of public has little or no direct interaction with the nursing staff and forms their perception of the nursing group indirectly through other ways, such as mass media (Fletcher, 2007; Girvin et al., 2016; Morris‐Thompson et al., 2011; Squires et al., 2019; Valiee et al., 2020). By combining public relations and literature descriptions, we considered mass media to have a special kind of public influence on people's image of nursing.

The public image of nursing is ultimately formed based on perceptions, experiences, and understanding of the nursing profession (Auker, 2004; López‐Verdugo et al., 2021; Maliheh et al., 2020; Mohsen et al., 2022; Squires et al., 2019). Perception is the interpretation of something created by people through their knowledge, experiences, attitudes and beliefs. Thus, perception is primarily a mental process (Glerean et al., 2017). Public perception refers to how people perceive nurses and nursing (Meiring & van Wyk, 2013; Donelan et al., 2008). Public perception invovles three key elements: cognition (Apaydin Cirik et al., 2022; Elmorshedy et al., 2020), emotion (Sahakyan et al., 2020) and behavioural intention (Daigle, 2018; Sahakyan et al., 2020; Thomas et al., 2010).

Cognition refers to the knowledge and beliefs shared by the public regarding nursing and its associated information, and it serves as the foundation for the public's perception of nursing. Multiple studies have shown that members of the public still maintain traditional stereotypes about nursing (Emeghebo, 2006; Hoeve et al., 2014; van der Cingel & Brouwer, 2021), with ambiguous public images of nursing, and display a lack of awareness of nursing functions, activities and roles (Emeghebo, 2006; Foà et al., 2021; Godsey et al., 2020; Hadid & Khatib, 2015; Kalisch et al., 2007; Roshangar et al., 2021). Emotion is a type of emotional response to nursing that represents a certain attitude of the public towards nursing and affects their overall assessment of nursing. The emotional reactions of the public towards nursing depend on the public's level of cognition regarding the profession. When the public has an inaccurate view of the nursing, the profession is easily misrepresented (Rodríguez‐Pérez et al., 2022). Mass media and the experience of the public when interacting with nurses are two important aspects that shape the public's feelings towards nursing. For example, negative images about nursing portrayed by mass media are more likely elicit negative attitudes towards nursing (McGillis Hall & Kashin, 2016; Şahan et al., 2021) and influence the public to view many aspects of nursing negatively (Meiring & van Wyk, 2013). Media praises in nursing feats is likely to influence viewers' positive perceptions of nursing (Cao et al., 2022). Squires et al. (2019) found a positive relationship between the nurses' attitudes towards caring for patients and their perceptions of care. Behavioural intention refers to the intention of the public to use care services, and it plays a crucial role in determining the behaviour that the public adopts regarding to nursing. Behavioural intentions are partly derived from emotional reactions (Thomas et al., 2010). Patients' feelings about nurses can influence their behaviours, such as their willingness to provide personal information during nursing evaluations and their willingness to receive educational and therapeutic interventions (Daigle, 2018; Thomas et al., 2010). Positive emotions may result in individuals exhibiting positive inclinations towards certain behaviours (Donelan et al., 2008). According to a survey on public opinions of nursing in the United States, nursing is considered a highly respected profession, and the public tends to endorse nursing as a career choice for students (Donelan et al., 2008). In contrast, negative emotions frequently result in poor behavioural intentions (Sahakyan et al., 2020) and mistrust in nurses' competence in carrying out nursing activities (Sahakyan et al., 2020). When the public holds negative attitudes towards nursing, they are sceptical of nurses' ability to care for patients (Sahakyan et al., 2020).

The public's perception of nursing relies heavily on the elements of cognition, emotion, and behavioural purposes. Cognitive information has a direct impact on the public's affective judgement, which in turn affects their behavioural choices. Public perception not only helps to depict the development process of the nursing public image, but also represents the outcomes of that image.

#### 3.3.3 Information (medium of interaction between the collective subject and the collective object)

Research shows that the information obtained by patients from their interaction with nurses and the portrayal of nurses in the media influences people's views on nursing (Ali et al., 2021; Donelan et al., 2008; Meiring & van Wyk, 2013; Şahan et al., 2021; Ward, 2006). This information exchange between nurses and the public forms a circulation system through which a public image of nursing is developed (Auker, 2004; Hallam, 1998). From the perspective of image generation, we conclude that subject‐object interaction is realised through information generation, dissemination, identification, processing and judgement.

Information generation reflects the objective basis for how nurses shape information. Information generation in nursing is a precondition for understanding nursing and the public impressions and views about it (Cabaniss, 2011; Cao et al., 2022; Gill & Baker, 2021; Wocial et al., 2018). Information dissemination is an important channel through which nursing professionals can connect with the public. Both information sources and media play important roles in the process of dissemination (Apaydin Cirik et al., 2022; Maliheh et al., 2020; Meiring & van Wyk, 2013; Morris‐Thompson et al., 2011; Rubbi et al., 2017; Ward, 2006; Weaver et al., 2014). We propose that mass media can be both the sender of information content and the medium of information dissemination. The media‐transmitted content can significantly affect the public's assessment of nursing information (Foà et al., 2021). Several complex psychological processes, such as information identification, processing and value judgement, mediate the interaction between the public and the nursing profession, and, hence, influence the formation of a public view of nursing or nurses (Rubbi et al., 2017; Squires et al., 2019; van der Cingel & Brouwer, 2021). Information identification, processing, and evaluation reflect the public's subjective initiative and creativity of the public. Therefore, the public image of nursing is built through a process of direct and indirect interaction between the public and nursing via the generation of nursing information and information dissemination, which influences the public's recognition, processing, and judgement of information (Ali et al., 2021; Gill & Baker, 2021; Girvin et al., 2016; Godsey et al., 2020; Maliheh et al., 2020). The public's recognition, processing and judgement of information play a leading role in the formation of the nursing image, which reflects the public's understanding and evaluation of nursing and is the core process in the formation of a nursing public image.

### 3.4 Antecedents

Antecedents are factors that affect conceptual attributes. This study included three antecedents: factors related to nursing, factors related to the public, and special events.

#### 3.4.1 Nursing‐related factors

Representative traditional nursing images mainly include handmaiden (Tzeng, 2006; Karanikola et al., 2011; Hadid & Khatib, 2015; van der Cingel & Brouwer, 2021; Lúanaigh, 2017), angels (Lúanaigh, 2017; Meiring & van Wyk, 2013) and sexy nurses (Lúanaigh, 2017; Kelly et al., 2012). The handmaiden embodies a male‐dominated patriarchal hierarchical structure that requires nurses to perform nursing work according to the requirements of doctors. This is based on the stereotype that nurses are physicians' handmaidens (van der Cingel & Brouwer, 2021). The angel image emphasizes the caring qualities of the nurse, such as caring for the patient and being empathetic towards the patient's daily struggles (van der Cingel & Brouwer, 2021). Sexy nurses embody romanticism, which often results in their treatment as sexual objects as seen in many pornographic films (Kelly et al., 2012). The formation of these images is closely related to the historical, social, religious, cultural, political and geographical contexts in which nursing has evolved (Ali et al., 2021; Grinberg & Sela, 2022; Hadid & Khatib, 2015; Meiring & van Wyk, 2013; Mohsen et al., 2022; Squires et al., 2019; Varaei et al., 2012), and is the main source of the public image of nursing (Hoeve et al., 2014; Maliheh et al., 2020; Morris‐Thompson et al., 2011). Although some traditional images of nurses have established positive images of nurses, such as the spirit of caring and dedication, most members of the public still view nursing as a low‐status occupation and nurses as less skilled professionals (Hadid & Khatib, 2015). These negative views continue to affect the development of the contemporary nursing profession (van der Cingel & Brouwer, 2021).

Gender is an important variable that influences the public image of nursing (Karanikola et al., 2011). Society generally considers nursing as a feminine profession (Apaydin Cirik et al., 2022; Cao et al., 2022; Elmorshedy et al., 2020; Gill & Baker, 2021; Glerean et al., 2017; Godsey et al., 2020). Linking femininity to care and compassion has become a central concept in nursing (Rezaei‐Adaryani et al., 2012; van der Cingel & Brouwer, 2021; Weaver et al., 2014). These gender role stereotypes influence public perceptions and discourage men from pursuing nursing as a profession (Squires et al., 2019). In addition, male nurses still face many obstacles in their work, such as gender discrimination and negative patient attitudes (Sharma et al., 2022). Consequently, the turnover rate of male nurses is twice that of female nurses (Valizadeh et al., 2014). Sharma et al. (2022) pointed out the proportion of registered male nurses in various regions of the world, 9.1% in America, 10.7% in Britain, 2%–15% in Europe, 7.8% in Ireland, 4.9% in Japan and 1% in China. These data indicate that the stereotype of a predominantly female profession which seriously limits the development of both the nursing profession and society.

The education level of nurses not only shapes the public image (Hadid & Khatib, 2015; Maliheh et al., 2020), but also affects the quality of health service delivery (Squires et al., 2019). The unequal level of entry requirements in nursing schools not only affects the public's perception of nursing but also nurses' perception of their own profession (Godsey et al., 2020; Rezaei‐Adaryani et al., 2012). Some British media have treated nursing as a profession with less rigorous educational training (Girvin et al., 2016). Nurse leaders and managers agree that higher levels of education will earn this profession more respect and trust (Sahakyan et al., 2020). Further, higher educational levels are associated with greater career development and have been shown to positively improve the overall image of nursing (Sahakyan et al., 2020).

Aspects such as nurses' uniforms, titles and occupational scope can also influence the public's perception of nursing (Auker, 2004; Daigle, 2018; Roshangar et al., 2021; Thomas et al., 2010; van der Cingel & Brouwer, 2021). For example, clothes, hairstyles and jewellery worn by the nurses can influence the patient–nurse interactions and therapeutic relationships (Thomas et al., 2010). Additionally, in a healthcare setting, when patients cannot distinguish nurses' uniforms from those of other assistant staff or when the name tag, title and qualifications are not clear, they are likely to perceive the nurse's role negatively (Godsey et al., 2020). In Iran, the terms ‘Parastar’ and ‘nurse’ are used interchangeably to designate any person responsible for providing any kind of care in a hospital or home (nursing assistant, person in charge of home care for children or older adults) (López‐Verdugo et al., 2021; Roshangar et al., 2021). Additionally, nurses often perform jobs outside their scope of practice, including the roles of physicians, laboratory technicians and pharmacists (Ndirangu et al., 2021; Roshangar et al., 2021). This scenario conveys inconsistent or incorrect messages that confuse and obscure the public perception, leading to an incorrect image of nursing (Godsey et al., 2020; López‐Verdugo et al., 2021).

The professional behaviour of the nurse, including providing care to the patient, determines the patient's experience of nursing care (Wocial et al., 2014), which also affects the nursing image (Emeghebo, 2006). In any profession, the negative behaviour or performance of some members can affect their perception of the entire profession (Glerean et al., 2019; Godsey et al., 2020; Morris‐Thompson et al., 2011; Valizadeh et al., 2014). Common examples include arguing in the workplace (Godsey et al., 2020), laziness at work (Appiah et al., 2020), inappropriate use of mobile phones during working hours and other similar unprofessional behaviour (Appiah et al., 2020), and replies like ‘ask your physician’ and ‘I don't know’ when a patient asks a nurse about their illness. Such actions lead patients to perceive the nurse as incompetent, and damage the public image of nursing (Valizadeh et al., 2014).

Nursing is the largest healthcare profession, however, nurses rarely participate in public discussions on the impact and value of their role in healthcare (Maliheh et al., 2020). In addition, nurses do not invest sufficient energy in creating and maintaining a positive professional image (Hoeve et al., 2014). Disunity among nurses has also contributes to a decline in their public image (Rezaei‐Adaryani et al., 2012).

#### 3.4.2 Public‐related factors

Many studies have suggested that the behaviour and language of professional colleagues as internal organizational public, such as hospital administrators and physicians, are most likely to affect the public image of care (Ali et al., 2021; Emeghebo, 2006; Foà et al., 2021; Girvin et al., 2016; Godsey et al., 2020; Valizadeh et al., 2014). A positive image of nursing by other health professionals promotes the better integration of nurses into multidisciplinary teams (Foà et al., 2021). In contrast, when physicians use demeaning comments in front of patients or other professionals or nurses, the image of nursing is damaged to some extent (Ali et al., 2021; Emeghebo, 2006; Girvin et al., 2016; Godsey et al., 2020; Valizadeh et al., 2014). In addition, the communication patterns of members of different professions can directly affect patient care and professional image (Apaydin Cirik et al., 2022; Ndirangu et al., 2021). Support from family members or friends and information about the nursing profession may influence young people's decisions to pursue nursing as a profession (Girvin et al., 2016; Glerean et al., 2017; Glerean et al., 2019; Mohsen et al., 2022; Morris‐Thompson et al., 2011; Varaei et al., 2012). When a nurse is a relative, they can creat a positive impression of nursing care for family members (Varaei et al., 2012). Interactions between caregivers, patients and families can enhance or improve the professional image of nursing (Hadid & Khatib, 2015; Rubbi et al., 2017). In particular, patients who receive care regular care have more opportunities to learn about other aspects of care and thus have a positive perception of care (Foà et al., 2021).

Mass media is one of the most important variables affecting the public image of nursing. The information conveyed by the media shapes people's attitudes and views about nursing (Auker, 2004; Cabaniss, 2011; Donelan et al., 2008; Foà et al., 2021; Glerean et al., 2017; Grinberg & Sela, 2022; Maliheh et al., 2020; Morris‐Thompson et al., 2011; Rezaei‐Adaryani et al., 2012; Rubbi et al., 2017; Şahan et al., 2021; Ward, 2006). This is particularly true in the absence of personal experience with nurses and/or healthcare providers. In such cases, the public's perception of care is largely derived from information obtained from television, novels, movies and the Internet (Price & McGillis Hall, 2014). When the media actively reports on the performance of care in emergencies, such as the fight against COVID‐19, it gains public praise (Cao et al., 2022). However, some media outlets ignore the contribution of nursing care to healthcare or present it unrealistically or unfavourably (Morris‐Thompson et al., 2011; Varaei et al., 2012; Lúanaigh, 2017). The media continues to reinforce stereotypes of nurses, such as angels, doctors' maids, battleaxes and sexy nurses (Hoeve et al., 2014; Karanikola et al., 2011; Kelly et al., 2012). In addition, negative events during nurses' service delivery are often highlighted in social media and newspapers (Girvin et al., 2016; McGillis Hall & Kashin, 2016), providing nurses with a professional image of failure and incompetence (Rodríguez‐Pérez et al., 2022). Inaccurate and misleading portrayals of nurses and the nursing profession in the media not only damage the public perception of nursing, but also seriously hinder the development of the nursing profession (Afshar et al., 2020; Gill & Baker, 2021; Godsey et al., 2020).

Individual knowledge levels play a role in the perception of nursing image (Cabaniss, 2011; Varaei et al., 2012). As reported by RoshanOAr et al. (2021), the ambiguous image of nurses among the Iranian public is due to their lack of awareness and knowledge of their nursing roles, responsibilities, and capabilities. Morris (2010) found that the public seemed to lack an understanding of the reality of nursing, including remuneration, the nature of nursing work, professional autonomy and the opportunities for clinical career promotion.

Personal values and preferences also affect every image and judgement in people's minds (Cabaniss, 2011; Maliheh et al., 2020; Valiee et al., 2020). In their analysis of nursing images, Kalisch et al. (2007) pointed out that the basic values acquired by all people in the process of socialisation affect the cognitive process of individuals, resulting in ingrained distorted knowledge and beliefs, including views on professional groups. People are unaware of the nurses' professional qualifications, expertise, and education. Instead, they give nurses a unified identity that includes being hardworking, low‐paid, conscientious, passive, oppressed, caring and female (Morris‐Thompson et al., 2011).

Multiple studies have demonstrated a link between the experiences of patients or family members receiving care and their image of nursing care (Donelan et al., 2008; Girvin et al., 2016; Godsey et al., 2020; Hadid & Khatib, 2015; Maliheh et al., 2020; Meiring & van Wyk, 2013; Ward, 2006). In Hadid and Khatib's (2015) study, respondents who received nursing care were more familiar with the job responsibilities of nurses and reported a better image of nursing. Girvin et al. (2016) revealed that respondents who received nursing services from nurse specialists expressed a more positive impression of nursing professionals. Nurses' attitudes towards patients (Squires et al., 2019) and the frequency with which patients and their families receive care (Hadid & Khatib, 2015; Woldasemayat et al., 2022; Godsey et al., 2020) affect the public's perception of the nursing profession.

#### 3.4.3 Special events

When nursing staff face major emergencies such as SARS, the Ebola virus, COVID‐19, the Indian tsunami and the Wenchuan earthquake, they make important contributions to alleviating human suffering (Godsey et al., 2020; McGillis Hall & Kashin, 2016; Rezaei‐Adaryani et al., 2012; Tzeng, 2006). These events give the public an opportunity to learn more about the efforts and capabilities of the nurses (Foà et al., 2021; Roshangar et al., 2021). Nurses are greatly respected by the public (Cao et al., 2022; Donelan et al., 2008; Şahan et al., 2021; van der Cingel & Brouwer, 2021; Zhang et al., 2021), have a positive impact on the public image of the nursing profession, and enhance the professional identity of nursing (Apaydin Cirik et al., 2022).

### 3.5 Consequences

This study included outcomes related to nursing and to the public.

#### 3.5.1 Consequences related to nursing

Individual aspects include self‐esteem/self‐concept (Karanikola et al., 2011; Takase et al., 2002; Zhang et al., 2021), professional identity (Hoeve et al., 2014; van der Cingel & Brouwer, 2021; Gill & Baker, 2021; López‐Verdugo et al., 2021; Cao et al., 2022; Kelly et al., 2012; Zhang et al., 2021; Lúanaigh, 2017), job performance (Afshar et al., 2020; López‐Verdugo et al., 2021; Rezaei‐Adaryani et al., 2012; Takase et al., 2002; Takase et al., 2006), job satisfaction (Afshar et al., 2020; Karanikola et al., 2011; Maliheh et al., 2020; Morris‐Thompson et al., 2011; Takase et al., 2002), work life quality (Roshangar et al., 2021) and poor renumeration (Karanikola et al., 2011; López‐Verdugo et al., 2021; Rezaei‐Adaryani et al., 2012; Varaei et al., 2012). Hoeve et al. (2014) discussed the relationship between nurses' public image, self‐concept and professional identity. Hallam (1998) believed that the public image of nursing affected nurses' self‐identity and professional pride. In constrast, Varaei et al. (2012) postulated that nursing image is closely related to the professional identity and responsibilities of nurses. Nurses obtain their self‐concept and professional identity from the unknown image of public nursing. Therefore, when a public nursing image presents an inaccurate description, it may damage nurses' professional social identity and status (Gill & Baker, 2021). Many studies have found that nurses' public image is positively correlated with job satisfaction, job performance, and quality of work. The more positive the public image of nursing, the higher the nurses' satisfaction, work ability and quality of life (Maliheh et al., 2020; Roshangar et al., 2021). Some studies suggest that the public image of nursing should not be closely related to financial rewards (López‐Verdugo et al., 2021), because when nurses are seen as angels, they should help others without expecting to be paid (Varaei et al., 2012).

The public image of nursing impacts care quality (Woldasemayat et al., 2022; Grinberg & Sela, 2022; Karanikola et al., 2011; Tzeng, 2006; Valiee et al., 2020; Varaei et al., 2012), staff shortage (Maliheh et al., 2020; Ndirangu et al., 2021; Rezaei‐Adaryani et al., 2012), interprofessional cooperation (Gill & Baker, 2021; Godsey et al., 2020), resources allocation (Girvin et al., 2016; Kalisch et al., 2007; Rezaei‐Adaryani et al., 2012) and industry development (Cabaniss, 2011; López‐Verdugo et al., 2021; Varaei et al., 2012). Numerous studies have found that the public image of nursing is positively correlated with the quality of care (López‐Verdugo et al., 2021; Valiee et al., 2020). Indeed, nurses have often reported that public criticism affects their ability to focus on work, which is a possible cause for mistakes. Many studies have demonstrated that a poor public image has also resulted in many secondary school students shunning away from choosing nursing as their major (Foà et al., 2021; Hadid & Khatib, 2015; Morris‐Thompson et al., 2011; Rubbi et al., 2017) and increased nurse turnover rates (Apaydin Cirik et al., 2022; Elmorshedy et al., 2020; Maliheh et al., 2020; Takase et al., 2006). This indicates that negative stereotypes about nursing affect the number of people willing to join the profession and service delivery by nurses (Auker, 2004; Hadid & Khatib, 2015; Hoeve et al., 2014; Karanikola et al., 2011; Meiring & van Wyk, 2013; Mohsen et al., 2022; Price & McGillis Hall, 2014). However, gender stereotypes discourage men from joining the nursing profession (Glerean et al., 2017; Roshangar et al., 2021; Weaver et al., 2014). In addition, studies have demonstrated that a poor professional image increases nurses' dissatisfaction at work (Grinberg & Sela, 2022; Valizadeh et al., 2014; Varaei et al., 2012), their intention to quit the profession, and the dropout rate of nursing students (Apaydin Cirik et al., 2022).

A few studies believe that the social image of nursing does not match the real situation of the nursing profession, resulting in conflicts in interprofessional cooperation (Godsey et al., 2020; Rezaei‐Adaryani et al., 2012). In addition, it affects resource allocation and decision‐making and hinders the expansion (Grinberg & Sela, 2022; Kalisch et al., 2007) and development of the profession (Sahakyan et al., 2020; van der Cingel & Brouwer, 2021; Zhang et al., 2021).

#### 3.5.2 Consequences related to the public

A positive public image of nursing reflects high‐quality care and can positively affect patient health (Cabaniss, 2011; Varaei et al., 2012). However, the poor public image of nursing discourages teamwork and fosters staff competition, leading to adverse patient outcomes (Godsey et al., 2020; Woldasemayat et al., 2022).

The public image of nursing is one of the most important factors affecting patient satisfaction (Rezaei‐Adaryani et al., 2012; Valiee et al., 2020). Nurses' professional behaviour and good communication can enhance patients' comfort and satisfaction (Maliheh et al., 2020). Woldasemavat et al. (2022) showed that when nursing care satisfaction increased by one point, patients' perceptions of their occupation increased by 1.769 times.

Several studies have found that the public image of nursing can directly affect the public's trust in and respect for the nursing profession (Gill & Baker, 2021; López‐Verdugo et al., 2021; Rezaei‐Adaryani et al., 2012). Recent studies have found that the COVID‐19 pandemic has made the public more aware of the nursing profession and changed their perceptions of nursing. In addition, the pandemic increased the trust the public has in nursing (Şahan et al., 2021).

As the largest community in the healthcare system, nurses plays an important role in ensuring the proper functioning of health services (Morris‐Thompson et al., 2011). Opposing views of the public towards the nursing profession hinders nurses from meeting the health needs of society (Roshangar et al., 2021), indirectly affecting the overall level of national health due to a shortage of nursing staff (Lúanaigh, 2017).

### 3.6 Surrogate and related term

Surrogate terms can be used interchangeably to express concepts. Related concepts are terms with common attributes and different characteristics from the original concepts (Rodgers & Knafl, 2000). The surrogate terms in this study included the social image of nursing (López‐Verdugo et al., 2021; Lusk, 2000) and nursing group images (Auker, 2004). Related concepts of public nursing image include nursing and professional nursing images. The related concepts had content similar to the public image of nursing. Rezaei‐Adaryani et al. (2012) stated that the nursing images includes the public image of nursing and described a broader conceptual category. The nursing professional image is derived from the perceived population, including nursing and non‐nursing staff. Therefore, the concept of nursing image is based on the impressions and perceptions of nursing. The core of the different concept names depends on the different populations that forms their perception.

## 4 DISCUSSION

This study clarified the attributes, antecedents, and consequences of the public image of nursing through concept analysis.

### 4.1 Analysis of attributes

#### 4.1.1 Analysis of nursing attributes

We identified nursing (nursing as the collective object), public (public as the collective object) and information (the medium of interaction between the collective subject and collective object) as attributes associated with the formation of a public image of nursing. Nursing elements consist of both intrinsic and extrinsic factors. Two scholars conducted conceptual analyses of nursing images. Ward (2006) believes that care, attitudes, knowledge, behaviour, autonomy, and nurse uniforms are the elements of the nursing image. Therefore, the author focuses on explaining public image of nursing from the one‐dimensional perspective of nursing connotations. The six elements of nursing image proposed by Ward frequently appear in literatures included in this study. These elements summarise the characteristics and attributes of nursing. However, with the multidirectional development of the nursing profession, these elements can no longer fully reflect the connotations of nursing. Therefore, this study classified the elements of nursing (objects) into professionalism, knowledge, and skills. Professionalism is the core element of nursing attributes and embodies the ideals, beliefs, value pursuits and moral norms shared by the entire nursing community. In addition to professionalism, nursing specialisation is the focus of nursing work (Afshar et al., 2020). This specialisation is based on the professional knowledge and skills of the nursing staff, which are also important in cultivating professionalism (Afshar et al., 2020). These three elements are interrelated and collectively reflect the intrinsic nursing characteristics. These characteristics are expressed through the specific behaviour, language and appearance of members of the profession when they are active in the field of nursing. Intrinsic and extrinsic factors are organically unified and collectively constitute the nursing dimensions.

Many studies have explored the perceptions and attitudes of nursing students towards the nursing profession. In these studies, nursing students were treated as part of the public population. This study concluded that the professional identity of nursing students and their performance, such as their comments on social media related to or unrelated to the profession, influenced their people's perceptions of nurses (De gagne et al., 2019). Therefore, as a nursing community subgroup, they are also an important part of the image of nursing.

#### 4.1.2 Analysis of public attributes

In their concept analysis, Rezaei‐Adaryani et al. (2012) proposed that nursing public image is a dimension of nursing image. However, this concept has not yet been analysed or explained further. In summary, we summarized the attributes of the public (subject), including public categories and public perceptions.

In previous studies, the classification of public categories varied. The various public categories were affected by individual experiences, values and cognitive levels because the studies reported different understandings of nursing images. Thus, from the perspective of public relations, communication and the above factors, a reasonable division was developed in this study based on close interactions between the public and nurses. Rezaei‐Adaryani et al. (2012) considered media images as another dimension of nursing images. They postulated that the mass media could be both public view and a medium tool from different aspects. However, this study argues that simply viewing mass media as a tool for information dissemination does not reveal the impact of media images on the nursing profession. As a result, the focus should be on the description of the care presented by the media, which is fabricated by media personnel or media organizations and represents their view of care (Squires et al., 2019). Media image is a processed and socialised product that reflects the demand for politics and power in contemporary society (Girvin et al., 2016). Media images of nursing and public opinion have a lasting and profound impact on the public, as they can change the public's perception of nursing. This study considers mass media as a public category.

The public constructs a subjective perception of nursing, recognising it as a subject to appraise and shape their understanding through a synthesis of objective facts, cognitive assessment, and emotional components.

This subjective reality is not a mirror reflection of objective reality but a comprehensive judgement made by the selective processing of objective things based on one's values. In addition, this subjective feature indicates that the quality of the experience of the public, judgement and evaluation of nursing are key factors in forming a positive or negative public image of nursing. In addition, public perception should consider individual and group factors. Individual factors imply that the attitudes and views of different individuals towards the same thing are always different because of differences in individual cognition, emotion, will and other factors. Individual perceptions are often susceptible to the influence of group perceptions, resulting in herd conformity.

#### 4.1.3 Analysis of information attributes

As an attribute of the nursing public image, information was analysed from the objective relationship between nursing and the public. Information is an essential element in image generation, as a medium for nursing and public interaction. Public perception of information is a process in which the public actively identifies, processes, and judges the transmitted information. Many studies have shown that the perception of the public regarding nursing care does not coincide with the practice of care (Foà et al., 2021; Hadid & Khatib, 2015; Hoeve et al., 2014; López‐Verdugo et al., 2021). This may be because of the authenticity (Foà et al., 2021) and completeness of the information (Girvin et al., 2016). Inaccurate representations in the media can lead to negative feelings towards the services offered by nurses to patients, other professionals, and society (Gill & Baker, 2021). Limited contact with nursing has contributes to the public's partial understanding of nursing (Godsey et al., 2020). The more nursing services the public receives, the higher the public knowledge about nursing, and the more positive the public perception of the nursing profession (Hadid & Khatib, 2015).

### 4.2 Analysis of antecedents and consequences

We selected two attributes of nursing (nursing as the collective object) and public (public as the collective subject), to determine the factors influencing the public image of the nursing profession. In recent decades, nursing practices have contributed to the improvement of social healthcare during various disaster events. This has increased the visibility and awareness of the nursing profession in several public events, such as health disasters. Additionally, the recent COVID‐19 pandemic has affected the public image of nursing. Poor public image of nursing results in negative outcomes during the provision of nursing services.

### 4.3 Limitations of the study

This study was performed based on studies published in English, suggesting that related research on the public image of nursing in other countries may be excluded. Research published in other languages could be useful in analysing and comparing public perceptions and evaluations of the public nursing image in different social and cultural contexts.

## 5 CONCLUSION

The public image of nursing is a sensitive indicator of the social status of the profession and has important political and economic significance. The COVID‐19 pandemic has helped scholars understand the need to conduct research on the public image of nursing. This study used Rogers' concept analysis to clarify the attributes, antecedents, and consequences of the public image of nursing. The results of this study provide a reference for developing corresponding promotional strategies in nursing practice, management and education.

## AUTHOR CONTRIBUTIONS

*Study design*: DY, FXQ. *Data collection*: DY. *Data analysis*: DY, FXQ. *Study supervision*: FXQ, XHY. *Manuscript writing*: DY. *Critical revisions for important intellectual content*: FXQ, XHY.

## FUNDING INFOMATION

None.

## CONFLICT OF INTEREST STATEMENT

The authors declare that they have no known competing financial interests or personal relationships that could have appeared to influence the work reported in this paper.

## ETHICS STATEMENT

Research Ethics Committee approva was approved by the Institutional Review Board of West China Hospital of Sichuan University [No: 2022(238)].

## Data Availability

The data that support the findings of this study are availability from the corresponding author upon reasonable request.

## APPENDIX 1 SEARCH STRATEGIES OF DATABASE

### 1.1

**Table:**

Database	Search formulation
PubMed	(nurs*[Title/Abstract]) AND (image [Title/Abstract])
CINHAL	(TI image OR AB image OR SU image) AND (TI nurs* OR AB nurs* OR SU nurs*)
Web of Science (WOS)	((TS= (nurs*)) OR TI = (nurs*) OR AB = (nurs*)) AND ((TS=(image) OR TI = (image)) OR AB = (image))
Scopus	TITLE‐ABS‐KEY(“nurs*”) AND TITLE‐ABS‐KEY(“image”)
ProQuest	(ti(nurs) OR ab(nurs*) OR su(nurs*)) AND (ti(image) OR ab(image) OR su(image))

*Note*: Extraction ‘English language’, ‘Source type’ and ‘Publication Date’.

## References

[nop270033-bib-0001] Afshar, L. , Ebadi, A. , Farmad, S. A. , & Azemian, A. (2020). Factors affecting the professional behavior of Iranian nurses: A qualitative study. Journal of Complementary Medicine Research, 11(3), 106–117. 10.5455/jcmr.2020.11.03.14

[nop270033-bib-0002] Albert, N. M. , Wocial, L. , Meyer, K. H. , Na, J. , & Trochelman, K. (2008). Impact of nurses' uniforms on patient and family perceptions of nurse professionalism. Applied Nursing, 21(4), 181–190. 10.1016/j.apnr.2007.04.008 18995159

[nop270033-bib-0003] Ali, I. , Bhutto, R. A. , Muhammad, S. , Ahmed, W. , Saeed, A. , & Saddiqui, S. (2021). Nursing profession–public image at private tertiary care hospital of Karachi. Medical Forum Monthly, 32(12), 34–37.

[nop270033-bib-0004] Apaydin Cirik, V. , Gül, U. , & Aksoy, B. (2022). The image of nursing among nursing and other healthcare professional university students: A mixed‐method study. Nurse Education in Practice, 59, 103293. 10.1016/j.nepr.2022.103293 35066255

[nop270033-bib-0005] Appiah, E. O. , Agyeiwaa, J. , & Amponsah, A. (2020). The changing public image of nursing in Ghana. Africa Journal of Nursing and Midwifery, 22(2), 1–13. 10.25159/2520-5293/7664

[nop270033-bib-0006] Auker, S. G. (2004). The image of the profession of nursing and its discursive representation in print media. Pennsylvania State University, Ph.D.

[nop270033-bib-0008] Cabaniss, R. (2011). Educating nurses to impact change in nursing's image. Teaching and Learning in Nursing, 6(3), 112–118. 10.1016/j.teln.2011.01.003

[nop270033-bib-0009] Cao, H. , Chen, Y. , He, X. , Song, Y. , Wang, Q. , & Yang, H. (2022). Chinese nurses' self‐expression media image during COVID‐19 pandemic: A qualitative media image analysis. Nursing Open, 9(2), 1164–1172. 10.1002/nop2.1156 35029042 PMC8859088

[nop270033-bib-0010] Chapman, C. M. (1977). Image of the nurse. International Nursing Review, 24, 166–170. 243499

[nop270033-bib-0011] Cohen, S. , & Bartholomew, K. (2008). Our image, our choice: Perspectives on shaping, empowering, and elevating the nursing profession. HCPro, Inc.

[nop270033-bib-0013] Daigle, A. (2018). Professional image and the nursing uniform. Journal of Continuing Education in Nursing, 49(12), 555–557. 10.3928/00220124-20181116-06 30496597

[nop270033-bib-0014] De gagne, J. C. , Hall, K. , Conklin, J. L. , Yamane, S. S. , Roth, N. W. , Chang, J. H. , & Kim, S. S. (2019). Uncovering cyberincivility among nurses and nursing students on twitter: A data mining study. International Journal of Nursing Studies, 89, 24–31. 10.1016/j.ijnurstu.2018.09.009 30321747

[nop270033-bib-0015] Donelan, K. , Buerhaus, P. , DesRoches, C. , Dittus, R. , & Dutwin, D. (2008). Public perceptions of nursing careers: The influence of the media and nursing shortages. Nursing Economic$, 26(3), 143–165. 18616051

[nop270033-bib-0016] Drenkard, K. , Swartwout, E. , & Hill, S. (2002). Nursing exploration summer camp: Improving the image of nursing. The Journal of Nursing Administration, 32(6), 354–362. 10.1097/00005110-200206000-00012 12055492

[nop270033-bib-0017] Elmorshedy, H. , AlAmrani, A. , Hassan, M. H. A. , Fayed, A. , & Albrecht, S. A. (2020). Contemporary public image of the nursing profession in Saudi Arabia. BMC Nursing, 19(1), 1–8. 10.1186/s12912-020-00442-w 32528229 PMC7285542

[nop270033-bib-0018] Emeghebo, L. E. (2006). Nurses' perceptions of the image of the profession of nursing. Teachers College, Columbia University.

[nop270033-bib-0019] Fealy, G. M. (2004). 'The good nurse': Visions and values in images of the nurse. Journal of Advanced Nursing, 46(6), 649–656. 10.1111/j.1365-2648.2004.03056.x 15154906

[nop270033-bib-0020] Fletcher, K. (2007). Image: Changing how women nurses think about themselves. Literature review. Journal of Advanced Nursing, 58(3), 207–215. 10.1111/j.1365-2648.2007.04285.x 17474909

[nop270033-bib-0021] Foà, C. , Bertuol, M. , Baronchelli, E. , Beltrami, G. , Toninelli, S. , Zamboni, L. , Artioli, G. , & Caruso, R. (2021). The influence of media representations on citizens' perceptions towards nurses: A comparison between before and after the Covid‐19 pandemic. Acta Biomed, 92(S2), e2021429. 10.23750/abm.v92iS2.12614 35037635

[nop270033-bib-0094] Geller, L. , & Summers, S. , 2014. Changing how the world thinks about nursing. Canadian Nurse, 110(1), 26–30. 24645383

[nop270033-bib-0022] Gill, J. , & Baker, C. (2021). The power of mass media and feminism in the evolution of nursing's image: A critical review of the literature and implications for nursing practice. Journal of Medical Humanities, 42(3), 371–386. 10.1007/s10912-019-09578-6 31713004

[nop270033-bib-0023] Girvin, J. , Jackson, D. , & Hutchinson, M. (2016). Contemporary public perceptions of nursing: A systematic review and narrative synthesis of the international research evidence. Journal of Nursing Management, 24(8), 994–1006. 10.1111/jonm.12413 27406529

[nop270033-bib-0024] Glerean, N. , Hupli, M. , Talman, K. , & Haavisto, E. (2017). Young peoples' perceptions of the nursing profession: An integrative review. Nurse Education Today, 57, 95–102. 10.1016/j.nedt.2017.07.008 28755570

[nop270033-bib-0025] Glerean, N. , Hupli, M. , Talman, K. , & Haavisto, E. (2019). Perception of nursing profession–focus group interview among applicants to nursing education. Scandinavian Journal of Caring Sciences, 33(2), 390–399. 10.1111/scs.12635 30604883

[nop270033-bib-0026] Godsey, J. A. , Houghton, D. M. , & Hayes, T. (2020). Registered nurse perceptions of factors contributing to the inconsistent brand image of the nursing profession. Nursing Outlook, 68(6), 808–821. 10.1016/j.outlook.2020.06.005 32763085 PMC7398865

[nop270033-bib-0027] Gomez, E. , & Brostoff, M. (2018). Helping high school students explore nursing careers in a summer internship program. Journal for Nurses in Professional Development, 34(3), 133–141. 10.1097/nnd.0000000000000446 29715205

[nop270033-bib-0028] Grinberg, K. , & Sela, Y. (2022). Perception of the image of the nursing profession and its relationship with quality of care. BMC Nursing, 21(1), 1–8. 10.1186/s12912-022-00830-4 35272645 PMC8908293

[nop270033-bib-0029] Hadid, S. , & Khatib, M. (2015). The public's perception of the status and image of the nursing profession. Medicine and Law, 34(1), 69–90. 30759925

[nop270033-bib-0030] Hall, L. M. , Angus, J. , Peter, E. , O'Brien‐Pallas, L. , Wynn, F. , & Donner, G. (2003). Media portrayal of Nurses' perspectives and concerns in the SARS crisis in Toronto. Journal of Nursing Scholarship, 35(3), 211–216. 10.1111/j.1547-5069.2003.00211.x 14562487 PMC7194222

[nop270033-bib-0032] Hallam, J. (1998). From angles to handmaidens: Changing constructions of nursing's public image in post‐war Britain. Nursing Inquiry, 5, 32–42. 9611579 10.1046/j.1440-1800.1998.510032.x

[nop270033-bib-0034] Hoeve, Y. , Jansen, G. , & Roodbol, P. (2014). The nursing profession: Public image, self‐concept and professional identity. A discussion paper. Journal of Advanced Nursing, 70(2), 295–309. 10.1111/jan.12177 23711235

[nop270033-bib-0035] Kalisch, B. J. , Begeny, S. , & Neumann, S. (2007). The image of the nurse on the internet. Nursing Outlook, 55(4), 182–188. 10.1016/j.outlook.2006.09.002 17678683

[nop270033-bib-0036] Kalisch, P. A. , & Kalisch, B. J. (1982a). Nurses on prime‐time television. The American Journal of Nursing, 82(2), 264–270. 6915710

[nop270033-bib-0037] Kalisch, P. A. , & Kalisch, B. J. (1982b). Nurses on prime‐time television. The American Journal of Nursing, 82(2), 1220–1224. 6915710

[nop270033-bib-0038] Kalisch, P. A. , & Kalisch, B. J. (1982c). Nurses on prime‐time television. The American Journal of Nursing, 82(2), 605–611. 6915710

[nop270033-bib-0039] Karanikola, M. N. , Papathanassoglou, E. D. , Nicolaou, C. , Koutroubas, A. , & Lemonidou, C. (2011). Greek intensive and emergency care nurses' perception of their public image: A phenomenological approach. Dimensions of Critical Care Nursing, 30(2), 108–116. 10.1097/DCC.0b013e3182052250 21307691

[nop270033-bib-0040] Kelly, J. , Fealy, G. M. , & Watson, R. (2012). The image of you: Constructing nursing identities in YouTube. Journal of Advanced Nursing, 68(8), 1804–1813. 10.1111/j.1365-2648.2011.05872.x 22070735

[nop270033-bib-0041] King, J. , Hardie, K. , & Conway, J. (2007). The perceptions of high school careers advisers regarding nursing: An Australian study. Contemporary Nurse, 24(2), 137–146. 10.5172/conu.2007.24.2.137 17563322

[nop270033-bib-0042] Koo, M. , & Lin, S. C. (2016). The image of nursing: A glimpse of the internet. Japan Journal of Nursing Science, 13(4), 496–501. 10.1111/jjns.12125 27162121

[nop270033-bib-0043] Kress, D. , Godack, C. A. , Berwanger, T. L. , & Davidson, P. M. (2018). The new script of nursing: Using social media and advances in communication–to create a contemporary image of nursing. Contemporary Nurse, 54(4‐5), 388–394. 10.1080/10376178.2018.1537720 30474519

[nop270033-bib-0044] Li, Q. G. , Hu, J. L. , Zheng, Y. W. , & Ruan, H. (2018). Concept analysis and its application in nursing. Journal of Nursing Science, 33(4), 100–102. 10.3870/j.issn.1001-4152.2018.04.100

[nop270033-bib-0045] López‐Verdugo, M. , Ponce‐Blandón, J. A. , López‐Narbona, F. J. , Romero‐Castillo, R. , & Guerra‐Martín, M. D. (2021). Social image of nursing. An Integrative Review about a Yet Unknown Profession. Nursing Reports, 11(2), 460–474. 10.3390/nursrep11020043 34968221 PMC8608107

[nop270033-bib-0093] Lúanaigh, P.Ó. , 2017. Nurses and nursing, The Person and the Profession. Routledge.

[nop270033-bib-0046] Lusk, B. (2000). Pretty and powerless: Nurses in advertisements, 1930–1950. Research in Nursing & Health, 23, 229–236. 10871538 10.1002/1098-240x(200006)23:3<229::aid-nur7>3.0.co;2-g

[nop270033-bib-0047] Maliheh, N. M. L. , Ashraf, A. , Hamid, H. M. , Taghi, S. M. , & Fatemeh, H. N. (2020). The public nursing image as perceived by nurses and citizens: A questionnaire survey. International Journal of Caring Sciences, 13(3), 1611–1620.

[nop270033-bib-0048] Marcinowicz, L. , Foley, M. , Zarzycka, D. , Chlabicz, S. , Windak, A. , & Buczkowski, K. (2009). Polish medical students' perceptions of the nursing profession: A cross‐sectional study. Scandinavian Journal of Caring Sciences, 23(3), 438–445. 10.1111/j.1471-6712.2008.00638.x 19000084

[nop270033-bib-0092] McGillis Hall, L. , Kashin, J. , 2016. Public understanding of the role of nurses during Ebola. Journal of Nursing Sholarship, 48(1), 91–97. 10.1111/jnu.12182 26642005

[nop270033-bib-0049] Meiring, A. , & van Wyk, N. C. (2013). The image of nurses and nursing as perceived by the south African public. Africa Journal of Nursing and Midwifery, 15(2), 3–15.

[nop270033-bib-0050] Mert, S. , Yildiz, T. A. , Senturk, S. G. , & Durualp, E. (2020). Senior high school students' opinions on the nursing profession: A ten‐year comparative study. Journal of Advanced Nursing, 76(8), 2082–2093. 10.1111/jan.14403 32350900

[nop270033-bib-0051] Milisen, K. , De Busser, T. , Kayaert, A. , Abraham, I. , & de Casterle, B. D. (2010). The evolving professional nursing self‐image of students in baccalaureate programs: A cross‐sectional survey. International Journal of Nursing Studies, 47(6), 688–698. 10.1016/j.ijnurstu.2009.11.008 19962697

[nop270033-bib-0052] Mohsen, A. , Bluvstein, I. , Wilf Miron, R. , & Kagan, I. (2022). Public image of the profession is associated with the choice of nursing career among Arab high school students: A cross‐sectional study. Journal of Nursing Management, 30(1), 310–317. 10.1111/jonm.13454 34414628

[nop270033-bib-0053] Morris, V. (2010). Nursing and nurses: The image and the reality. Nursing Management, 17(1), 16–19. 10.7748/nm2010.04.17.1.16.c7642 20432640

[nop270033-bib-0054] Morris‐Thompson, T. , Shepherd, J. , Plata, R. , & Marks‐Maran, D. (2011). Diversity, fulfilment and privilege: The image of nursing. Journal of Nursing Management, 19(5), 683–692. 10.1111/j.1365-2834.2011.01268.x 21749542

[nop270033-bib-0055] Murphy, F. , Jones, S. , Edwards, M. , James, J. , & Mayer, A. (2009). The impact of nurse education on the caring behaviours of nursing students. Nurse Education Today, 29, 254–264. 10.1016/j.nedt.2008.08.016 18945526

[nop270033-bib-0056] Ndirangu, E. W. , Sarki, A. M. , Mbekenga, C. , & Edwards, G. (2021). Professional image of nursing and midwifery in East Africa: An exploratory analysis. BMC Nursing, 20(1), 1–11. 10.1186/s12912-020-00531-w 33676509 PMC7936462

[nop270033-bib-0057] Neilson, G. R. , & McNally, J. (2013). The negative influence of significant others on high academic achieving school pupils' choice of nursing as a career. Nurse Education Today, 33(3), 205–209. 10.1016/j.nedt.2012.02.019 22464633

[nop270033-bib-0058] Price, S. L. , & McGillis Hall, L. (2014). The history of nurse imagery and the implications for recruitment: A discussion paper. Journal of Advanced Nursing, 70(7), 1502–1509. 10.1111/jan.12289 24224541

[nop270033-bib-0059] Price, S. L. , McGillis Hall, L. , Angus, J. E. , & Peter, E. (2013). Choosing nursing as a career: A narrative analysis of millennial nurses' career choice of virtue. Nursing Inquiry, 20(4), 305–316. 10.1111/nin.12027 23551958

[nop270033-bib-0060] Rahman, R. M. A. E. , & Shousha, A. A. E. F. (2013). Perceptions of the public image of nursing among baccalaureate nursing students. Life Science Journal, 10(12s), 1061–1071.

[nop270033-bib-0061] Rezaei‐Adaryani, M. , Salsali, M. , & Mohammadi, E. (2012). Nursing image: An evolutionary concept analysis. Contemporary Nurse, 43(1), 81–89. 10.5172/conu.2012.43.1.81 23343236

[nop270033-bib-0062] Rodgers, B. L. , & Knafl, K. A. (2000). Concept development in nursing: Foundations, techniques, and applications (pp. 77–102). Saunders.

[nop270033-bib-0063] Rodríguez‐Pérez, M. , Mena‐Navarro, F. , Domínguez‐Pichardo, A. , & Teresa‐Morales, C. (2022). Current social perception of and value attached to nursing professionals' competences: An integrative review. International Journal of Environmental Research and Public Health, 19(3), 2–19. 10.3390/ijerph19031817 PMC8834898 35162838

[nop270033-bib-0064] Roshangar, F. , Soheil, A. , Moghbeli, G. , Wiseman, T. , Feizollahzadeh, H. , & Gilani, N. (2021). Iranian nurses' perception of the public image of nursing and its association with their quality of working life. Nursing Open, 8(6), 3441–3451. 10.1002/nop2.892 33951343 PMC8510743

[nop270033-bib-0065] Rubbi, I. , Cremonini, V. , Artioli, G. , Lenzini, A. , Talenti, I. , Caponnetto, V. , La Cerra, C. , Petrucci, C. , & Lancia, L. (2017). The public perception of nurses. An Italian cross‐sectional study. Acta Biomed, 88(5s), 31–38. 10.23750/abm.v88i5-S.6884 PMC6357583 29189703

[nop270033-bib-0066] Rubbi, I. , Pasquinelli, G. , Cremonini, V. , Fortunato, F. , gatti, L. , Lepanto, F. , Artioli, G. , & Bonacaro, A. (2019). Does student orientation improve nursing image and positively influence the enrolment of nursing students in the university? An observational study. Acta Biomed, 90(6s), 68–77. 10.23750/abm.v90i6-S.8568 PMC6776179 31292417

[nop270033-bib-0067] Sahakyan, S. , Akopyan, K. , & Petrosyan, V. (2020). Nurses role, importance and status in Armenia: A mixed method study. Journal of Nursing Management, 28(7), 1561–1569. 10.1111/jonm.13109 32715532

[nop270033-bib-0068] Şahan, S. , Yıldız, A. S. , & Ergin, E. (2021). A review of public perceptions about nurses communicated via twitter in Turkey. Public Health Nursing, 1‐5, 638–642. 10.1111/phn.12999 34706103

[nop270033-bib-0069] Sharma, S. K. , Mudgal, S. K. , Rawat, R. , Sehrawat, S. , Mehra, T. , & Choudhary, S. (2022). Patient perception towards males in nursing profession in India: A single center, cross‐sectional survey. International Journal of Africa Nursing Sciences, 16, 100417. 10.1016/j.ijans.2022.100417

[nop270033-bib-0070] Simmons, L. W. (1962). Past and potential: Images of the nurse. Nursing Forum, 1, 16–33.

[nop270033-bib-0071] Skorupski, V. J. , & Rea, R. E. (2006). Patients' perceptions of today's nursing attire. The Journal of Nursing Administration, 36(9), 393–401. 16969250 10.1097/00005110-200609000-00005

[nop270033-bib-0072] Somers, M. J. , Finch, L. , & Birnbaum, D. (2010). Marketing nursing as a profession: Integrated marketing strategies to address the nursing shortage. Health Marketing Quarterly, 27(3), 291–306. 10.1080/07359683.2010.495306 20706896

[nop270033-bib-0073] Squires, A. , Ojemeni, M. T. , Olson, E. , & Uchanieshvili, M. (2019). Nursing's public image in the republic of Georgia: A qualitative, exploratory study. Nursing Inquiry, 26(4), e12295. 10.1111/nin.12295 31637805

[nop270033-bib-0074] Stanley, D. J. (2008). Celluloid angels: A research study of nurses in feature films 1900–2007. Journal of Advanced Nursing, 64(1), 84–95. 10.1111/j.1365-2648.2008.04793.x 18808595

[nop270033-bib-0075] Takase, M. , Kershaw, E. , & Burt, L. (2002). Does public image of nurses matter? Journal of Professional Nursing, 18(4), 196–205. 10.1053/jpnu.2002.127014 12244538

[nop270033-bib-0076] Takase, M. , Maude, P. , & Manias, E. (2006). Impact of the perceived public image of nursing on nurses' work behaviour. Journal of Advanced Nursing, 53(3), 333–343. 10.1111/j.1365-2648.2006.03729.x 16441539

[nop270033-bib-0077] Thomas, C. M. , Ehret, A. , Ellis, B. , Colon‐Shoop, S. , Linton, J. , & Metz, S. (2010). Perception of nurse caring, skills, and knowledge based on appearance. The Journal of Nursing Administration, 40(11), 489–497. 10.1097/NNA.0b013e3181f88b48 20978418

[nop270033-bib-0078] Toren, O. , Kerzman, H. , & Kagan, I. (2011). The difference between professional image and job satisfaction of nurses who studied in a post‐basic education program and nurses with generic education: A questionnaire survey. Journal of Professional Nursing, 27(1), 28–34. 10.1016/j.profnurs.2010.09.003 21272833

[nop270033-bib-0079] Tzeng, H. M. (2006). Testing a conceptual model of the image of nursing in Taiwan. International Journal of Nursing Studies, 43(6), 755–765. 10.1016/j.ijnurstu.2005.10.004 16309686

[nop270033-bib-0080] Valiee, S. , Nemati, S. M. , & Valian, D. (2020). Exploration of service recipients' image of a perfect nurse: A qualitative descriptive study. Applied Nursing Research, 54, 151272. 10.1016/j.apnr.2020.151272 32650893

[nop270033-bib-0081] Valizadeh, L. , Zamanzadeh, V. , Fooladi, M. M. , Azadi, A. , Negarandeh, R. , & Monadi, M. (2014). The image of nursing, as perceived by Iranian male nurses. Nursing & Health Sciences, 16(3), 307–313. 10.1111/nhs.12101 24636021

[nop270033-bib-0082] van der Cingel, M. , & Brouwer, J. (2021). What makes a nurse today? A debate on the nursing professional identity and its need for change. Nursing Philosophy, 22(2), e12343. 10.1111/nup.12343 33450124

[nop270033-bib-0083] Varaei, S. , Vaismoradi, M. , Jasper, M. , & Faghihzadeh, S. (2012). Iranian nurses self‐perception–factors influencing nursing image. Journal of Nursing Management, 20(4), 551–560. 10.1111/j.1365-2834.2012.01397.x 22591156

[nop270033-bib-0084] Ward, J. (2006). The differences between public and nurses' perception of the image of registered nurses. Widener University School of Nursing.

[nop270033-bib-0085] Weaver, R. , Ferguson, C. , Wilbourn, M. , & Salamonson, Y. (2014). Men in nursing on television: Exposing and reinforcing stereotypes. Journal of Advanced Nursing, 70(4), 833–842. 10.1111/jan.12244 24001311

[nop270033-bib-0086] Weaver, R. , Salamonson, Y. , Koch, J. , & Jackson, D. (2013). Nursing on television: Student perceptions of television's role in public image, recruitment and education. Journal of Advanced Nursing, 69(12), 2635–2643. 10.1111/jan.12148 23566250

[nop270033-bib-0087] Williams, C. , & Dickstein‐Fischer, L. (2019). School counselors' perceptions of necessary attributes of middle and high school students interested in a career in nursing. Nursing Education Perspectives, 40(1), 30–34. 10.1097/01.Nep.0000000000000348 29994887

[nop270033-bib-0088] Williams, C. , Dickstein‐Fischer, L. , & Emery, H. (2018). A call for future nurses: School counselors' perceptions of the nursing role: A preliminary study. Journal of Professional Nursing, 34(1), 54–59. 10.1016/j.profnurs.2017.07.004 29406140

[nop270033-bib-0089] Wocial, L. D. , Sego, K. , Rager, C. , Laubersheimer, S. , & Everett, L. Q. (2014). Image is more than a uniform. The Journal of Nursing Administration, 44(5), 298–302. 10.1097/NNA.0000000000000070 24759203

[nop270033-bib-0007] Woldasemayat, A. L. , Mekonnen Zeru, L. , & Demissie Abathun, A. (2022). Perception towards nursing profession and associated factors among patients at Jimma Medical Center, Ethiopia. A cross‐sectional study. International Journal of Africa Nursing Sciences, 17, 100445. 10.1016/j.ijans.2022.100445

[nop270033-bib-0091] Zhang, Z. , Fu, W. , Tian, C. , Zhang, F. , Zhao, B. , Mao, J. , & Saligan, L. N. (2021). Professional identity of Chinese nursing students during the COVID‐19 pandemic outbreak: A nation‐wide cross‐sectional study. Nurse Education in Practice, 52, 103040. 10.1016/j.nepr.2021.103040 33813343 PMC9760126

